# Giant bullous emphysema mistaken for traumatic pneumothorax

**DOI:** 10.1016/j.ijscr.2019.02.005

**Published:** 2019-02-13

**Authors:** Edson Gonçalves Ferreira Junior, Philippos Apolinario Costa, Larissa Melo Freire Golveia Silveira, Luis Enrique Maurera Almeida, Nayane Carolina Pertile Salvioni, Bruna Menon Loureiro

**Affiliations:** Universidade Federal do Vale do São Francisco, Av. José de Sá Maniçoba, S/N - Centro CEP: 56304-917, Petrolina, PE, Brazil

**Keywords:** Giant bullous emphysema, Traumatic pneumothorax, Vanishing lung, Bullectomy

## Abstract

Introduction: Giant bullous emphysema (GBE) is defined by giant bullae in one or both upper lobes, occupying at least one-third of the hemithorax and compressing the surrounding parenchyma [1]. Symptoms include dyspnea, hypoxia, chest pain and pressure, and hemoptysis [2], which can be complicated by pneumothorax and infection of the bullae [3].

Case presentation: A 50-year-old male was brought to the emergency department after he fell 5 m in a suicide attempt. The patient was in respiratory distress and had bilateral absence of breath sounds. He was intubated and bilateral chest tubes were inserted. A computerized tomography (CT) scan showed bilateral giant bullous emphysema in the upper lobes, confirming a diagnosis of GBE.

As a result of the insertion of chest tubes, he developed bilateral high flow fistulas. During his hospitalization, he developed sepsis secondary to ventilator-associated pneumonia. In an attempt to control the fistulas, a right bullectomy was performed. Despite antibiotic treatment and surgical intervention, the patient died due to septic shock.

Discussion: The clinical picture of a patient with GBE can be similar to that of pneumothorax, and GBE has been reported as being misdiagnosed as pneumothorax [4,5]. A CT scan can play an important role in differentiating these conditions [6], thus avoiding needle decompression, which can be catastrophic [6].

Conclusion: Giant bullous emphysema can represent a pitfall in trauma assessment. We recommend that in cases where pneumothorax is suspected, if the patient is clinically stable, imaging studies should be performed prior to chest tube placement.

## Highlights

• The authors present a unique case of giant bullous emphysema in the context of a trauma evaluation.
• During initial trauma evaluation giant bullous emphysema can be misdiagnosed as pneumothorax.
• A computerized tomography scan can avoid catastrophic complications in patients with giant bullous emphysema, such as uncontrollable airway fistulas.

## 1 Introduction

Giant bullous emphysema (GBE), also known as type I bullous disease or vanishing lung syndrome, is a condition that was first described by Burk in 1937 [7]. Fifty years after Burk’s initial description, Roberts et al. established the radiographic criteria for this syndrome as the presence of giant bullae in one or both upper lobes, occupying at least one-third of the hemithorax and compressing the normal surrounding parenchyma [1]. The bullae are usually asymmetric and occur predominantly in the upper lobes [8].

This is described as a progressive disease in which bullae do not participate in oxygenation, often leading to dyspnea, hypoxia, symptomatic chest pain and pressure, hemoptysis, and spontaneous pneumothorax. GBE is typically seen in young, thin, male smokers. The clinical syndrome has been associated with cigarettes, inhaled drug abuse, a-1 antitrypsin deficiency, Marfan syndrome, and Ehlers-Danlos syndrome [2].

Chronic obstructive pulmonary disease (COPD)-related emphysematous bullae are the most common types that lead to GBE [9]. With the progression of COPD, the obstruction increases in severity and eventually becomes irreversible [10].

GBE can be complicated by pneumothorax and infection of the bullae [3].

In GBE, pneumothorax can be provoked by mechanical ventilation, recurrent infections, and even malignancy [11].

Our objective is to present a case of GBE in the context of initial trauma evaluation. This report follows the SCARE guidelines [12].

## 2 Case presentation

A 50-year-old male was brought to the emergency department after he jumped from a 5-meter bridge in an attempted suicide and fell on the hard concrete below. Upon admission, the patient was agitated, disoriented, and in intense respiratory distress. Examination revealted that the patient’s airway was clear, but there was a bilateral absence of breath sounds and hyperresonance on percussion. The patient was hemodynamically stable. He was intubated due to respiratory failure. Bilateral chest tubes were inserted based on a high clinical suspicion of pneumothorax. Thereafter, the patient developed a large subcutaneous emphysema, despite the fact that the chest tubes were functioning with his severe air leakage. Past medical history was unremarkable with no previous formal depression diagnosis. A social history check showed daily marijuana and tobacco use.

**Fig. 1:**
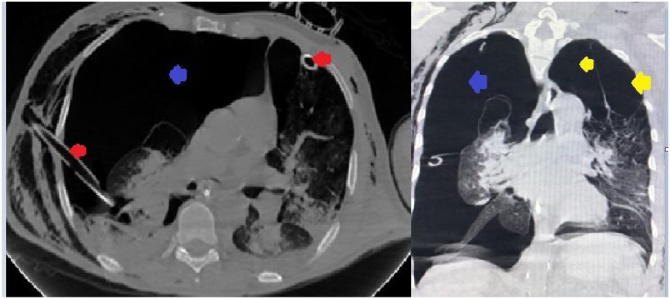
Thorax CT without contrast demonstrates subcutaneous emphysema, right lobe lung collapse, and absence of pneumothorax on the left side. Red arrows indicate chest tubes. Blue arrows indicate pneumothorax. Yellow arrows indicate bullae.

**Fig. 2:**
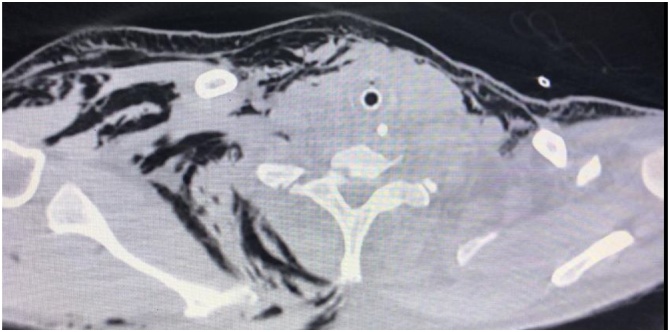
Thorax CT without contrast demonstrated subcutaneous emphysema after chest tube placement.

The patient was sent for a head, neck, thorax, abdomen, and pelvis CT scan. The scan revealed giant bullous emphysema on the superior lobes bilaterally, right pneumothorax with a collapsed lung, along with multiple rib fractures, and lung emphysema (Fig. 1, Fig. 2). A hip dislocation was detected, and closed reduction was performed.

The patient was sent to the ICU, where he improved clinically after conservative treatment with continuous negative pressure suction using a 20 cm water column. He was extubated after 48 h, with persistence of the air leakage on both chest tubes.

On the day following extubation, he developed hypoxia associated with disorientation and agitation and had to be intubated again. Hypoxia was postulated from pulmonary contusion and ventilator-associated pneumonia worsening his already baseline compromised lung. He developed sepsis, and subsequently acute kidney injury with the need for dialysis.

During the course of 5 days, the patient presented hypoxia and a severe mixed metabolic and respiratory acidosis, despite the use vancomycin and piperacillin/tazobactam. The treatment with bilateral chest tubes associated with continuous negative pressure aspiration did not correct the air leakage, which caused an important lost of tidal volumes on the ventilator. Changes in ventilator parameters (increases in PEEP, tidal volumes, etc.) did not improve his oxygenation or decrease his pCO2. In an effort to expand his lungs and improve his ventilatory function, we decided to perform a bilateral bullectomy.

**Fig. 3:**
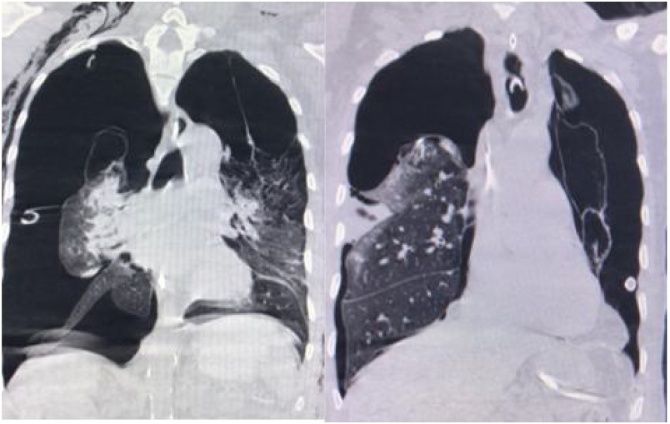
Thorax CT without contrast performed at admission on the left side, and one day after bullectomy on the right side. It demonstrates a significant improvement in pulmonary expansion after decortication of right lobe; the collapsed left side, however, was not demonstrated at time of admission to the trauma center, in a left image.

**Fig. 4:**
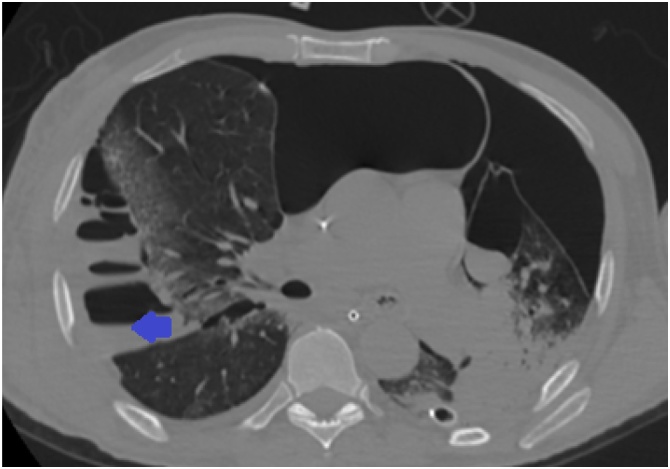
Thorax CT without contrast, bullectomy post-operative. The blue arrow indicates empyema and incarceration.

**Fig. 5:**
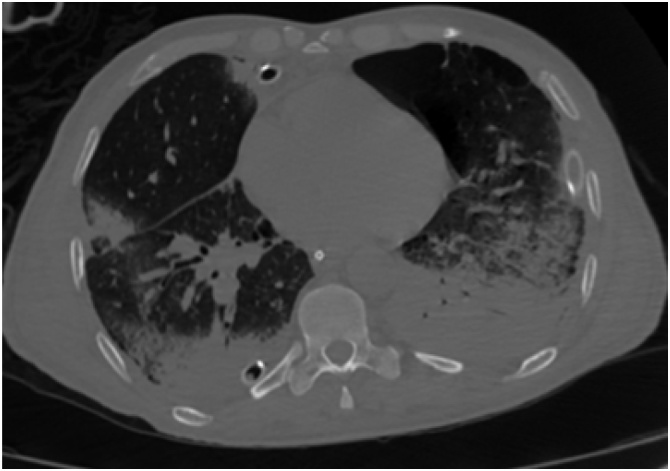
Thorax CT without contrast demonstrating a bilateral pulmonary consolidation despite pulmonary expansion.

As the patient had poor surgical status, only a right bullectomy was performed 8 days after the trauma. The right side was chosen over the left, because it showed more compression and a larger residual, healthier parenchyma. After surgery, the right side fistula was resolved (Fig. 3). Four days after surgery, the patient developed a right-sided empyema, right lung incarceration, and blood clots in the chest tube (Fig. 4). In order to resolve these issues, videothoracoscopy and decortication of the right lung were performed, which resulted in the resolution of these problems (Fig. 5) and improvement in ventilatory parameters with conservative treatment for the left lung. Notwithstanding antibiotic treatment and successful surgical interventions, the patient died on the 25^th^ day of hospitalization due to infectious complications.

## 3 Discussion

In trauma assessment, the steps of the primary survey are summarized by the mnemonic ABCDE (airway, breathing, circulation/hemorrhage, disability, and exposure/environment) [13].

Breathing determines the patient’s ability to ventilate and oxygenate. Critical findings include the absence of spontaneous ventilation, absent or asymmetric breath sounds (consistent with either pneumothorax or endotracheal tube malposition), dyspnea, hyperresonance (pneumothorax) or dullness (hemothorax) on chest percussion, and gross chest wall instability or defects that compromise ventilation (e.g., flail chest, sucking chest wound).

Tension pneumothorax is a clinical diagnosis reflecting air under pressure in the affected pleural space. Treatment should not be delayed in waiting for radiologic confirmation [13].

Advanced trauma life support (ATLS) also distinguishes simple pneumothorax from tension pneumothorax. Simple pneumothorax can be diagnosed in the secondary survey, with the help of imaging studies [13].

The reported patient was brought to the emergency department after a severe trauma. He had respiratory failure and signs that could indicate tension pneumothorax. For this reason he was intubated, and, based on the clinical suspicion of a bilateral tension pneumothorax, chest tubes were inserted.

GBE is defined as the presence of giant bullae in one or both upper lobes, which occupy at least one-third of the hemithorax and which compress the normal surrounding parenchyma [1]. Several risk factors had been described, but marijuana smoking seems to play an important role in the development of the disease. This is also correlated with early development and severe forms of the disease [14].

Dyspnea, hypoxia, chest pain, and pressure are signs and symptoms reported for GBE [2]. Upon physical examination of the first patient described with GBE, the chest was of the emphysematous type and hyperresonant on both sides during percussion. Breath sounds were absent except over the bases [7].

The clinical picture of a patient with GBE can be very similar to that of pneumothorax, and not infrequently these conditions can co-exist, as pneumothorax can be a complication of GBE [4]. On more than one occasion, GBE has been reported as having been misdiagnosed as pneumothorax [4,5].

To illustrate this, we created a table that shows other case reports and symptoms of GBE, risk factors, association with pneumothorax, and the treatment performed (Table 1).

**Table 1:** XXX.

CASE REPORT	SEX	AGE	SYMPTOMS	RISK FACTORS	PNEUMOTHORAX	TREATMENT
BALLAY ET AL 2017 [8]	WOMAN	50	DYSPNEA, CHEST PAIN	TOBACCO	?	SPIRATION IBV VALVES
JAISWAL ET AL 2017 [18]	WOMAN	48	DECREASED EXERCISE TOLERANCE	TOBACCO	N	BULLECTOMY
SAEED ET AL 2017 [19]	MAN	56	DYSPNEA, CHEST PAIN	TOBACCO, CANNABIS, AIDS	Y	HEIMLICH VALVE
SHARMA ET AL 2017 [3]	MAN	44	DYSPNEA, CHEST PAIN	TOBACCO, CANNABIS	N	CLINICAL
WIESEL ET AL 2017 [22]	MAN	47	DYSPNEA, COUGH, FEVER	TOBACCO, CANNABIS	N	CLINICAL
ANILE ET AL 2016 [17]	MAN	14	DYSPNEA, CHEST PAIN	NONE	N	BULLECTOMY
YUNHEE ET AL 2016 [5]	MAN	55	DYSPNEA, COUGH	TOBACCO	N	CLINICAL
CHEN ET AL 2015 [21]	WOMAN	58	DYSPNEA, COUGH	NONE	N	BULLECTOMY
HUANG ET AL 2014 [10]	MAN	59	DYSPNEA, COUGH	NONE	N	BULLECTOMY
LIANG ET AL 2014 [16]	MAN	35	DYSPNEA, CHEST PAIN, COUGH	TOBACCO	N	BULLECTOMY
LADIZINKI ET AL 2014 [20]	WOMAN	41	DYSPNEA, CHEST PAIN, COUGH	TOBACCO	N	?
VAN BAEL ET AL 2014 [11]	MAN	36	DYSPNEA, CHEST PAIN	TOBACCO	N	BULLECTOMY
WANG ET AL 2014 [15]	WOMAN	19	CHEST PAIN	TOBACCO	N	BULLECTOMY
KHASAWNEH ET AL 2013 [4]	MAN	65	DYSPNEA	TOBACCO, CANNABIS, AIDS	N	BULLECTOMY
YU-TZU TSAO AND SHIH-WEI LEE 2012 [6]	MAN	44	DYSPNEA, CHEST PAIN, COLD SWEATS	TOBACCO	Y	CHEST TUBE
**PRESENT CASE**	**MAN**	**50**	**DYSPNEA, CHEST PAIN**	**TOBACCO, CANNABIS**	**Y**	**CHEST TUBE AND BULLECTOMY**

In this case, since there was an absence of an imaging study before the chest tube placement, it is hard to differentiate traumatic pneumothorax from iatrogenic pneumothorax. Signs that might suggest the latter include the appearance of a subcutaneous emphysema immediately after the placement of chest tubes and the worsening of the left lung during his hospitalization (Fig. 3).

Chest radiography may help differentiate large bulla from pneumothorax. In GBE cases, the whole parenchyma is inferiorly compressed in the direction of the costophrenic angle, whereas pneumothorax presents itself as a lung parenchyma, which collapses into a clump toward the hilum [15].

It is of the utmost importance to differentiate between these two, given that needle decompression can be catastrophic [6]. The insertion of a chest tube or needles into a giant bulla can cause pneumothorax and uncontrollable air fistula.

One may, arguably, try to differentiate GBE from pneumothorax based only on the signs, symptoms, and chest radiography. However, chest radiography and physical examination cannot provide exact details on the precise anatomy of the findings. Thus, in this context, chest tomography (CT) is more suited for the adequate study of the pleural space and lungs [6]. A CT also can show any coexisting disease and complications [11].

One published case presented symptoms on the right side of the thorax that appeared to be related to a pneumothorax, when the pneumothorax was, in fact, on the left and changes in examination were due to severe compromise of the right side by GBE. Clarification was made possible by tomography [6].

For all the reasons stated above, inserting chest tubes based only on clinical suspicion must be reserved for clinically unstable patients [2]. All others would benefit from further investigations with a chest tomography.

In our case, chest tubes were inserted, in accordance with standard of care, without any imaging study due to respiratory instability. This resulted in iatrogenic airway fistulas that were not controlled with negative pressure tubes. Despite control of the fistulas by the bullectomy, the patient had a poor lung reserve function compared with his baseline condition. In this case, trauma, pulmonary contusion, and infection were determinant on his unfavorable outcome. In similar situations, ventilatory limitation due to rib fractures, pulmonary contusions, hemothorax, and pneumothorax, may overlap GBE, making diagnosis and treatment more challenging.

The treatment of choice for GBE is a limited bullectomy [6,16,17]. Generally this resection has no effect on lung function. The resection can be performed by video-assisted thoracoscopy or open procedure [4,11,18]. Other options, for example lung transplantation, have been reported in severe cases where resection is not an option, due to a large portion of the lung parenchyma being compromised [8,19].

In another case report, uncontrollable bronchopleural fistula, secondary to chest tube drainage of a suspected pneumothorax in a COPD patient, was treated with pneumonectomy, associated with an intrabronchial umbrella-shaped unidirectional valve deployed by flexible bronchoscopy. The outcome of the proposed treatment was satisfactory in this case, which is the only one to describe this type of treatment [8].

## 4 Conclusion

GBE is a little known rare entity, and as such there exist few available studies on the topic. It is a condition with a high associated mortality due to frequent association with severe pulmonary diseases. The usual treatment of GBE consists of bullectomy and smoking cessation; however, in some cases lung transplantation is the therapeutic option.

Even in non-traumatic cases, it is a diagnosis that frequently leads to misperception. Thus, during initial trauma evaluation, where time is of the essence and the team has limited knowledge of the patient’s past medical history, GBE can easily be misdiagnosed as pneumothorax.

We recommend that in patients who have suspected pneumothorax, if they are clinically stable, imaging studies should be performed prior to any chest tube insertion. Performing CT scans on these patients can avoid catastrophic complications, such as uncontrollable airway fistulas and death.

## Conflicts of interest

None.

## Funding

Authors did not receive any funding for this work.

## Ethical approval

We do not require ethical approval to write a case report paper.

## Consent

Written informed consent was obtained from the patient’s family for publication of this case report and accompanying images. A copy of the written consent is available for review by the Editor-in-Chief of this journal on request.

## Author contribution

1 Edson Gonçalves Ferreira Junior: Conceptualization, Methodology, Resources, Writing the paper, Writing – Review & Editing, Project Administration, Final approval
2 Philippos Apolinario Costa: Conceptualization, Methodology, Data collection, Data analysis/interpretation, Writing – Review & Editing, Final approval
3 Larissa de Melo Freire Gouveia Silveira: Conceptualization, Methodology, Data collection, Resources, Writing – Review & Editing, Final approval
4 Luis Enrique Maurera Almeida: Conceptualization, Methodology, Data collection, Resources, Writing – Review & Editing, Final approval
5 Nayane Carolina Pertile Salvioni: Conceptualization, Methodology, Data collection, Resources, Writing – Review & Editing, Final approval
6 6Bruna Menon Loureiro: Conceptualization, Methodology, Data collection, Resources, Writing – Review & Editing, Final approval

## Registration of research studies

Case reports don’t need to be registered.

## Guarantor

Edson Gonçalves Ferreira Junior.

## Provenance and peer review

Not commissioned, externally peer-reviewed.

## References

[1] Roberts L., Putman C.E., Chen J.T. (1987). Vanishing lung syndrome: upper lobe bullous pneumopathy. Rev. Interam. Radiol..

[2] Stern E.J., Webb W.R., Weinacker A., Muller N.L. (1994). Idiopathic giant bullous emphysema (vanishing lung syndrome): imaging findings in nine patients. AJR.

[3] Sharma N., Justaniah A.M., Kanne J.P., Gurney J.W., Mohammed T.L.H. (2009). Vanishing lung syndrome (giant bullous emphysema): CT findings in 7 patients and a literature review. J. Thorac. Imaging.

[4] Khasawneh F.A., Nakhla E.N., Karim A., Halloush R.A. (2013). Vanishing lung syndrome mistaken for bilateral spontaneous. pneumothorax. BMJ Case Rep..

[5] Im Yunhee, Farooqi Saad, Mora Adan (2016). Vanishing lung syndrome. Proc. (Bayl. Univ. Med. Cent)..

[6] Tsao Yu-Tzu, Lee Shih-Wei (2012). Vanishing lung syndrome. CMAJ.

[7] Burke R. (1937). Vanishing lungs: a case report of bullous emphysema. Radiology.

[8] Ballay N., Soder B., Smith J., Miller A., Headrick J.R. (2017). Intrabronchial pneumonectomy for vanishing lung syndrome: first reported case. Ann. Thorac. Surg..

[9] Ruan S.Y., Huang C.T., Chien J.Y. (2011). Non-surgical management of giant lung bullae during mechanical ventilation. Respir. Care.

[10] Huang W., Han R., Li L., He Y. (2014). Surgery for giant emphysematous bullae: case report and a short literature review. J. Thorac. Dis..

[11] Van Bael K., La Meir M., Vanoverbeke H. (2014). Video-assisted thoracoscopic resection of a giant bulla in vanishing lung syndrome: case report and a short literature review. J. Cardiothorac. Surg..

[12] Agha R.A., Fowler A.J., Saetta A., Barai I., Rajmohan S., Orgill D.P., for the SCARE Group (2016). The SCARE statement: consensus-based surgical case report guidelines. Int. J. Surg..

[13] American College of Surgeons (2012). Advanced Trauma Life Support Program for Physicians.

[14] Tashtoush B., Gonzalez-Ibarra F., Memarpour R., Hadeh A., Smolley L. (2014). Vanishing lung syndrome in a patient with HIV infection and heavy marijuana use. Case Rep. Pulmonol..

[15] Wang J., Liu W. (2014). Vanishing lung syndrome. Can. Respir. J..

[16] Liang J.J., Wigle D.A., Midthun D.E. (2014). Vanishing lung syndrome (Idiopathic giant bullous emphysema). Am. J. Med. Sci..

[17] Anile M., Diso D., Onorati I., Mantovani S., Venuta F. (2016). Unilateral vanishing lung syndrome. Thorax.

[18] Jaiswal P., Sreenivasan J., Jaiswal R. (2017). The vanishing lung. Postgrad. Med. J..

[19] Saeed S., Gray S. (2017). Vanishing lung syndrome in a patient with HIV infection and recurrent pneumothorax. Pan Afr. Med. J..

[20] Ladizinski B., Sankey C. (2014). Images in clinical medicine. Vanishing lung syndrome. N. Engl. J. Med..

[21] Chen H., Wang W., Feng J., Mei Y. (2015). Giant bullous emphysema in the right middle lobe. Int. J. Clin. Exp. Med..

[22] Wiesel S., Siddiqui F., Khan T. (2017). Vanishing lung syndrome: compound effect of tobacco and marijuana use on the development of bullous lung disease—a joint effort. Cureus.

